# Ethical and Legal Considerations in Posthumous Oocyte Retrieval for Minor Girls

**DOI:** 10.3390/ijerph23070865

**Published:** 2026-07-02

**Authors:** Anushka Chalmeti, Jason Lesandrini, David S. Reis

**Affiliations:** Wellstar Health Systems, 1800 Parkway Pl, Marietta, GA 30067, USA

**Keywords:** posthumous reproduction, posthumous oocyte retrieval, minors, assisted reproductive technology, medical ethics, reproductive law

## Abstract

**Highlights:**

**Public health relevance—How does this work relate to a public health issue?**

- Posthumous oocyte retrieval is an emerging reproductive technology that is raising clinical, legal, and ethical challenges for healthcare systems.
- Requests involving posthumous oocyte retrieval in minors create a unique dilemma for bodily autonomy, consent, and reproductive justice.

**Public health significance—Why is this work of significance to public health?**

- Existing clinical protocols, legal precedents, and ethical recommendations only address posthumous sperm retrieval in adult males, leaving significant gaps involving oocyte retrieval and procedures involving minors.
- Issuing clinical guidelines and legal standards can help protect minor girls and provide clarifications for clinicians, reducing stressful, end-of-life decision-making.

**Public health implications—What are the key implications or messages for practitioners, policy makers and/or researchers in public health?**

- Posthumous oocyte retrieval in minor girls should be disallowed.
- Healthcare institutions, professional societies, and lawmakers should issue guidance, recommendations, and legislation that explicitly ban posthumous oocyte retrieval in minor girls.

**Abstract:**

Posthumous gamete retrieval has emerged as a complex issue at the intersection of reproductive technology, bioethics, consent, and law. While posthumous sperm retrieval has received small amounts of legal, ethical, and clinical attention, posthumous oocyte retrieval, particularly in minors, remains largely unaddressed. This review examines the clinical, legal, and ethical considerations surrounding posthumous oocyte retrieval in minor girls. First, the paper explores the medical processes involved in posthumous gamete retrieval, drawing a significant distinction between sperm and oocyte retrieval. Second, it reviews international legal standards and U.S. jurisprudence relevant to posthumous reproduction, including both constitutional reproductive rights cases and court decisions directly addressing posthumous gamete use at the state level. Third, it analyzes existing institutional and professional guidelines governing posthumous reproduction in the United States. Across these three domains, the analysis reveals three main takeaways. First, current frameworks overwhelmingly assume adult decedents and focus primarily on sperm retrieval requested by spouses or partners, leaving a large unaddressed gap for minors and posthumous oocyte retrieval. Second, posthumous oocyte retrieval in minors presents distinct challenges related to medical burden, consent, parental authority, and the upbringing and welfare of potential offspring. Lastly, institutional, professional, and legislative guidance that prohibits posthumous oocyte retrieval in minors should be issued to provide clarity and recommendations for clinicians confronted with such requests.

## 1. Introduction

Maya Rodriguez, a 17-year-old high school senior, was declared dead by neurological criteria following a motor vehicle accident on her way home from a college visit. Her parents, both in their early fifties, arrived at the hospital within hours of the accident. After receiving the devastating news and understanding that their daughter would not recover, they made an unexpected request of the clinical ethics team: they wanted to pursue posthumous oocyte retrieval.

Mrs. Rodriguez explained that Maya had always been clear about wanting to be a mother. She recalled how Maya had recently told her mother that she “couldn’t wait to have kids someday” and had even chosen names she loved. Mr. Rodriguez added that Maya had a serious boyfriend, David, 18 years old, who had just started college at a nearby university. The parents had already spoken with David’s family, who they said were supportive and had identified a woman willing to serve as a gestational carrier. The Rodriguezes emphasized their commitment to raising any resulting child. “We know this sounds complicated,” Mr. Rodriguez acknowledged. “But Maya wanted to be a mother more than anything. We want to give her that chance, even though she’s gone. A child would be her legacy, and we would love and raise that baby as our grandchild.”

The clinical team explained that unlike sperm retrieval, oocyte retrieval would require keeping Maya on somatic support after determination of death by neurological criteria (hereafter referred to as “somatic support”) for an extended period—potentially weeks—to administer hormonal stimulation for oocyte maturation. The oocytes would then need to be retrieved through a transvaginal procedure, fertilized with David’s sperm, and transferred to a gestational carrier. The process would be medically complex, invasive, and require substantial resources and time.

The hospital had no formal policy addressing posthumous gamete retrieval requests from parents of minors, particularly for oocyte retrieval. Maya was an organ donor, and her organs were scheduled for retrieval within 48 h. The ethics consult service faced pressing questions: Should the hospital honor this request? Should Maya’s body be kept on somatic support for weeks to facilitate oocyte maturation? Who would make decisions about embryo disposition, gestational carriers, and the future child’s welfare?

This case reflects the complex intersection of grief, reproductive technology, and bodily autonomy that clinicians increasingly face. While posthumous sperm retrieval has generated some legal precedent and clinical guidance, posthumous oocyte retrieval in minor girls remains almost entirely unaddressed. The medical complexity, extended timeline, invasiveness of the procedure, and need for gestational carriers introduce layers of ethical and legal consideration that existing frameworks—built primarily around adult cases with spousal requests—are inadequate to address.

From a public health perspective, beyond individual ethical dilemmas, these cases raise questions of resource allocation, standardization of care provision, and the protection of vulnerable populations within healthcare systems. As emerging reproductive technologies advance past existing policy frameworks, the absence of guidance can create variability that may lead to inequitable and ethically inconsistent health outcomes.

Hence, this paper will attempt to address this gap in the literature. First, we outline the process of posthumous gamete and oocyte extraction to elucidate the complexities involved in the procedure. Second, we explore international and national legal jurisprudence, followed by an analysis of national healthcare guidelines. Third, we construct an argument against allowing posthumous oocyte retrieval in minor girls. Finally, we propose institutional guidelines for managing posthumous oocyte retrieval cases for minor girls.

## 2. Methodology

This study employed a narrative review methodology to examine the ethical, legal, and clinical considerations surrounding posthumous oocyte retrieval in minors. Sources were identified through searches conducted between 16 October 2025 and 13 November 2025 using PubMed and Google Scholar. Search terms included “posthumous reproduction”, “posthumous sperm retrieval”, “posthumous gamete retrieval”, “posthumous egg retrieval”, and “posthumous oocyte retrieval”. Given the incredibly limited amount of literature addressing posthumous oocyte retrieval, no publication date restrictions were identified. Additional relevant sources were identified through review of references cited within retrieved sources.

Sources included peer-reviewed literature; legal scholarship; international policy documents and analyses; domestic judicial opinions, statutes, and policy analyses; and professional society guidelines. Sources were selected based on their relevance to the study (including, but not limited to, legal questions answered, precedents in place, adjacent clinical guidelines, and existing industry frameworks). Sources that did not address posthumous reproduction or did not contribute substantively to the ethical, legal, or clinical questions examined in this study were excluded. Additionally, this review relied on English-language literature and translated or secondary descriptions of relevant legal authorities that were in other languages. The international jurisdictions discussed are illustrative rather than comprehensive. They were selected because they provide contrasting legal approaches and are frequently referenced in the posthumous reproduction literature.

Retrieved sources were reviewed and synthesized using thematic analysis: all sources were examined for common ethical, legal, and policy themes, including bodily autonomy, consent, parental decision-making authority, and reproductive intent. Areas of consensus, disagreement, and regulatory gaps were then identified, with particular attention to the lack of guidance regarding posthumous oocyte retrieval in minors.

## 3. Posthumous Gamete Retrieval Process

Posthumous gamete retrieval is a process in which oocytes or sperm are retrieved after a person is declared dead by neurological criteria. Regardless of the gamete being retrieved, posthumous retrieval is a complicated process that requires many considerations beyond medical feasibility alone. These include determining who has legal authority to make the request, establishing evidence of the deceased’s reproductive intentions, navigating institutional policies that vary widely across healthcare systems, securing appropriate storage facilities, and addressing questions of financial responsibility for both retrieval and long-term cryopreservation. Additionally, requestors must consider the psychological implications of delayed parenthood, the legal status of any resulting children, and the disposition of genetic material if it ultimately goes unused. Each of these considerations becomes more complex when the deceased is a minor who could not have provided legally valid consent or binding directives about reproductive intent.

The medical procedures themselves differ substantially between sperm and oocyte retrieval, introducing distinct ethical considerations. When retrieving sperm, the medical process is relatively straightforward and minimally invasive. Sperm can be obtained through epididymal aspiration (extracting fluid from the tube where sperm mature), percutaneous testicular biopsy (using a needle to extract tissue), or surgical removal of the testes [1]. The procedure typically takes less than an hour and must be completed within 36 h after death to ensure sperm viability [2]. The deceased’s body is not subjected to extended artificial support, hormonal manipulation, or repeated interventions.

Retrieving oocytes is substantially more involved and places significantly different demands on the deceased’s body. The process requires maintaining the body on artificial support—sometimes for weeks—while administering daily hormonal injections of gonadotropins to stimulate the ovaries and promote oocyte maturation. This hormonal stimulation mimics the process used in living patients undergoing in vitro fertilization but must be carefully managed in a brain-dead patient whose body no longer regulates these processes naturally. Medical teams must monitor hormone levels, adjust medications, and perform ultrasounds to track follicle development. Once oocytes reach maturity, they are collected through transvaginal ultrasound-guided aspiration, a procedure that involves inserting a needle through the vaginal wall to retrieve oocytes from the ovarian follicles [3]. Due to the complications regarding oocyte maturation and viability, the timeline for extraction varies considerably but can extend from one to four weeks depending on where the deceased was in her menstrual cycle at the time of death and how her body responds to stimulation [4].

The need for a gestational carrier after posthumous oocyte retrieval further compounds the medical, ethical, and legal dilemmas. Unlike posthumous sperm retrieval, where a living partner can carry the pregnancy herself, posthumous oocyte retrieval requires identifying a surrogate willing to undergo embryo transfer and carry the pregnancy to term. This introduces additional parties into the decision-making process, raises questions about compensation and legal parentage, and extends the timeline from retrieval to potential birth by many months or even years.

These procedural differences raise distinct moral questions. While some might argue that harms to a deceased person’s body should be weighed differently than harms to the living—since the deceased cannot experience pain, suffer indignity, or have their autonomy violated in the same way—the extended use of a deceased minor’s body for reproductive purposes nevertheless warrants careful ethical scrutiny. The degree of bodily invasion, the duration of intervention, and the transformation of the body into what might be characterized as a biological resource all raise questions about respect for the deceased, the limits of parental authority, and the purposes for which medicine should maintain bodies after brain death. Given these differences in medical complexity, time sensitivity, invasiveness, and the extended corporeal demands placed on the deceased, posthumous oocyte retrieval must be evaluated with greater ethical scrutiny and more robust procedural safeguards than those developed primarily for posthumous sperm retrieval. These differences also carry public health implications: prolonged use of intensive care resources for non-therapeutic purposes raises concerns about equitable allocation in an already resource-constrained healthcare setting. Standardizing how such cases are approached is, therefore, not only an ethical concern but a systems-level priority.

To advance this evaluation, an international jurisprudence perspective can provide insight into how different legal systems have confronted some of these challenges and offer diverse comparative frameworks for resolution.

## 4. International Jurisprudence

To understand the ethical and legal landscape surrounding posthumous gamete retrieval, it is useful to first examine how other countries have approached this issue through legislation and case law. International approaches vary widely, ranging from complete prohibition to regulated permission with specific consent requirements. However, as the following cases demonstrate, nearly all international frameworks focus primarily on posthumous sperm retrieval in adult cases where a spouse makes the request. The question of posthumous oocyte retrieval in minors remains largely unaddressed in international law, creating a gap in guidance precisely where the ethical stakes are highest; this global inconsistency shows the lack of shared standards governing emerging reproductive technologies, creating potential for divides in cross-border reproductive practices and exacerbating inequities in access, regulation, and oversight. However, the absence of direct precedent does not diminish the value of drawing upon existing jurisprudence and analogous cases to inform ethically grounded responses in cases involving minors.

### 4.1. France: Complete Ban

*Parpalaix v. CECOS* is the landmark legislation in France. When diagnosed with testicular cancer, Alain Parpalaix deposited sperm at the Centre d’Etude et de Conservation du Sperme (CECOS) in 1981. At the time of deposit, he was unmarried to his partner; as his cancer worsened, Alain and Corinne Parpalaix got married. Alain Parpalaix died two days later. Corrine then requested Alain’s sperm deposit, but CECOS refused because there was no written instruction from Alain about posthumous use, so he may not have wanted his spouse to use it for insemination. Corinne and her in-laws decided to pursue legal action. Due to inheritance reasons and verbal testimony that the sperm was deposited for insemination purposes [5], the Courts decided Alain’s parents had the right to ascertain Alain’s intent and they ultimately gave the sperm to Corinne. Following this, CECOS implemented a policy prohibiting the use of stored sperm for posthumous reproduction; this policy was later upheld by French courts, and France subsequently enacted legislation formally banning posthumous reproduction nationwide. Similar bans exist in Germany, Sweden, and Taiwan [6].

### 4.2. Spain: Regulated Access

Posthumous reproduction is regulated and legal in Spain. In fact, people from other countries often visit Spain when seeking posthumous insemination using a deceased partner’s sperm. LHART, Spain’s Assisted Reproduction Law, governs such reproduction by two decrees: if a man dies before his sperm has been used for impregnation, then (i) the man is not the legal parent of any child conceived using his sperm without special legal permission; unless (ii) if a man explicitly gives written consent before death—if so, his wife (or another specified woman) may use it for assisted reproduction within 12 months of his death. Additionally, if assisted reproduction began before death, the law assumes that he consented to it continuing [7].

However, this law is vague in addressing posthumous oocyte retrieval. Since it explicitly forbids the possibility of a deceased man consenting to insemination with genetic material of another man, doubts arise about transferring a pre-embryo (i.e., if oocytes are retrieved and fertilized with a living partner’s sperm to transfer into a surrogate). This pre-embryo transfer limitation also makes it illegal for same-sex couples in Spain to use posthumous assisted reproductive technologies [7].

### 4.3. England: Explicit Written Consent

English law, similar to Spanish law, allows posthumous reproduction with explicit written consent. Landmark legislation is England is the *R v. Human Fertilisation and Embryology Authority (HFEA), ex parte Blood* case. Stephen Blood orally expressed his desire to have children with his wife, Diane Blood, before falling into a coma due to his meningitis. Though the doctors let Diane retrieve Stephen’s sperm, they did not let her inseminate herself because she did not have Stephen’s written consent. Since such in vitro fertilization procedures were legal in Belgium, Diane asked to have the sperm moved to Belgium: this was not allowed at first by the HFEA, but the Court of Appeals later overturned their decision and allowed her to move the sperm to another country. Following this, the HFEA passed laws to extend the written consent requirement to the retrieval of sperm from patients who are comatose, and posthumous reproduction remains reserved only to those with valid, written consent of the deceased partner [6,8].

### 4.4. Israel: Implied Consent

Israel is an outlier in posthumous reproductive legalization with a comparatively relaxed and pronatalist stance [9,10]. Israeli posthumous reproduction policy is not codified and is instead outlined in guidelines written by the Attorney General. There are two main limitations on posthumous reproduction in these guidelines: (i) as long as consent was implied, a widow (any other party must obtain a court order) may retrieve her husband’s sperm, and (ii) a court will determine on a case-by-case basis to ensure that the deceased demonstrated an intent to become a parent before insemination [6,10].

As seen, there is a varied range of international jurisprudence regarding posthumous reproduction. Aside from Israel, almost every other country has formalized protocol for or a complete ban on posthumous reproduction, yet nearly all laws focus on posthumous sperm retrieval, with little to no attention to posthumous oocyte retrieval. In fact, the only reference to posthumous oocyte retrieval in Spain’s law is through an implied restriction of consent to a different individual’s genetic material. It is also important to note that the countries allowing posthumous reproduction mandate donors to be married—or at least older than 18—as the laws concern either a spousal request or written authorization, which cannot be granted without parental permission for minors.

## 5. National Jurisprudence

In the absence of explicit federal legislation addressing posthumous reproduction, courts have approached these cases by first establishing whether reproductive rights exist and second, determining how those rights apply when gametes are retrieved or used after death. The Supreme Court has never directly addressed posthumous reproduction, but lower courts deciding such cases have relied on broader reproductive rights jurisprudence to inform their reasoning. This section first examines the constitutional landscape of reproductive rights that courts have invoked when considering posthumous reproduction requests, then analyzes cases directly addressing posthumous gamete retrieval to understand the current legal boundaries and gaps in this area.

### 5.1. Constitutional Framework: Reproductive Rights Precedent

While none of these cases directly address posthumous reproduction, lower courts have cited them when considering posthumous retrieval requests. In *Meyer v. Nebraska* (1923), the Supreme Court ruled that a Nebraska law prohibiting grade schools from teaching their students any language other than English was unconstitutional. In their decision, they said that “[liberty] denotes not merely freedom from bodily restraint but also the right of the individual… to marry, establish a home and bring up children” [11]. If the right to liberty encapsulates a right to bring up children, then, as some legal scholars have argued, this can extend to decisions surrounding whether, when, and how to have them [6]—including through posthumous reproduction. In a similar vein, the Supreme Court argued in *Skinner v. Oklahoma ex rel. Williamson* (1942) that a Nebraska law allowing the sterilization of a person who had been convicted three or more times was unconstitutional. In this decision, SCOTUS declared that the Nebraska law “deprive[d] certain individuals of a right which is basic to the perpetuation of a race—the right to have offspring.” They further said that “procreation [is] fundamental” [12], making it a basic right for the American people. If the right to procreation is fundamental to an individual, then, as some legal scholars suggest, it can extend to posthumous reproduction [6].

Though *Roe* and *Casey* were overruled by *Dobbs*, their framing is important not only to analyze the legal penumbra of “liberty” that existed—and that this paper draws from—but also to understand lower court decisions that will be discussed later in this paper. To be clear, this analysis examines both existing reproductive rights jurisprudence and historical approaches to questions of reproductive autonomy and decision-making; the *Roe* and *Casey* precedents do not directly govern posthumous reproduction—rather, they are used to consider how existing legal frameworks may inform future judicial review of unaddressed issues in the reproductive area. In the famous *Roe v. Wade* (1971) and *Planned Parenthood of Southeastern Pennsylvania v. Casey* (1992) cases, the Court touched on reproductive rights [13,14]. In *Roe*, the Supreme Court ruled that a Texas law prohibiting abortion unless deemed as necessary by a doctor to save a pregnant person’s life was unconstitutional, stating that the right to privacy as found in the Ninth and Fourteenth Amendments must “encompass a woman’s decision whether or not to terminate her pregnancy” [13]. If a pregnant person can decide when to have children, it can be argued that this right can extend to cases of posthumous reproduction as well. In *Casey*, the Supreme Court ruled that a Pennsylvania rule requiring women who want an abortion to obtain informed consent, wait 24 h, and then if married, notify their husbands, and, if minors, obtain parental consent was unconstitutional. In this decision, the Supreme Court upheld *Roe*, declaring that “there is a realm of personal liberty which the government may not enter,” and that there exists “constitutional protection to personal decisions relating to marriage,” including “procreation, contraception, family relationships, child rearing, and education” They frame these choices as central to “personal dignity and autonomy [and] the liberty protected by the Fourteenth Amendment” [14]. Posthumous reproduction can, ergo, fall under the same umbrella of protected personal decisions within the family.

### 5.2. State Framework: Reproductive Rights Precedent

On the State level in *Lifchez v. Hartigan* (1990), the Illinois District Court was tasked to look at the legality of a subsection of Illinois’s abortion act that states “no person shall sell or experiment upon a fetus” made by artificial fertilization. The court decided that this law was unconstitutional, stating that “it takes no great leap of logic to see that within the cluster of constitutionally protected choices that includes the right to have access to contraceptives, there must be included within that cluster the right to submit to a medical procedure that may bring about, rather than prevent, pregnancy” [15]. A right to conceive can be extrapolated to include posthumous oocyte retrieval and reproduction, too [6].

In an Ohio case, *Cameron v. Board of Education of Hillsboro* (1991), a District Court looked at a case of sex discrimination where a teacher stated that the Board of Education discriminated against her because she conceived a child through artificial insemination and because of her status as an unwed mother. The court cited the Equal Protection Clause in its decision, saying that “A woman has a constitutional privacy right to control her reproductive functions. Consequently, a woman possesses the right to become pregnant by artificial insemination” [16]. This right to pregnancy through artificial insemination can also extend to insemination or oocyte retrieval posthumously [6].

Finally, in *Davis v. Davis* (1992), the Supreme Court of Tennessee took up the issue of “ownership of embryos [17]. In this case, Mary Sue Davis and Junior Davis were attempting to have children through IVF. When the couple initially started depositing sperm and retrieving oocytes for IVF, they were married. All six attempts to conceive through IVF failed, and the couple decided to wait one year before trying again, cryopreserving the unused embryos. However, during this year they got divorced and had not written a prior agreement regarding what should happen to the pre-embryos in case of separation. Post-divorce, Mary Sue wanted to keep trying to conceive with the pre-embryos, while Junior preferred them to remain cryopreserved. In September 1989, the trial court ruled that Mary Sue had custody of the pre-embryos and had discretion over future implantation. The Court of Appeals, on Junior’s appeal, reversed this decision, finding Junior had a constitutional right not to become a parent, and granted them joint custody. Appealed to the Supreme Court of Tennessee by Mary Sue, this case ultimately was forced to look at the legal status of pre-embryos—deciding whether they should be considered people, property, or a new designation. The Court cited *Roe* for arguing that federal law does not recognize the unborn as people; furthermore, the pre-embryos were even less developed than the ‘viability’ limit in abortion law. The decision states that with IVF processes, couples must have a written agreement about the procedure in case of sudden change, and that parties may be permitted to modify their initial IVF contracts only by mutual agreement [17]. This decision may pervade into posthumous reproductive spaces in the United States by requiring written spousal consent for such processes and mandating joint agreement in case of any unexpected challenges. Such restrictions will especially apply when a partner has a terminal illness and undergoes IVF to conceive a child in hopes of recovery; if this partner dies, the living spouse may not have the right to proceed with embryo implantations or surrogacy without prior written consent.

### 5.3. Direct Posthumous Reproduction Cases

While the constitutional cases above provide a framework that courts have referenced when considering reproductive rights more broadly, only a handful of cases have directly addressed posthumous gamete retrieval and use. These cases reveal both emerging legal patterns and significant gaps in jurisprudence. The three most instructive cases—Hecht, Christy, and Zhu—share several characteristics: all involve posthumous sperm retrieval, all concern male decedents who were adults or near-adults, and all required courts to balance evidence of the deceased’s reproductive intentions against competing interests such as family wishes or public policy concerns. Notably absent from this case law is any precedent addressing posthumous oocyte retrieval or cases involving minor decedents, leaving courts and clinicians with little guidance precisely where the ethical and legal complexities are greatest.

*Hecht v. Superior Court (Kane)* (1993) took place in California and disputed the legal discretion of sperm disposal. When William Kane deposited sperm into a sperm bank, he agreed with the bank that it would store or release the sperm depending on the wishes of the executor of his estate. Kane died by suicide almost one month later, and his estate’s executor was his girlfriend of five years, Deborah Hecht. His will left the sperm at the discretion of Hecht if she wished, but Kane’s adult children requested that the sperm be destroyed, claiming that posthumous conception was against public policy. The trial court granted the children’s request for sperm destruction, but Hecht appealed and won. The appellate court vacated the lower court’s order and declared that only the probate court could make decisions regarding sperm destruction after death [18,19]. The appellate court also declared that posthumous reproduction is not banned under public policy, saying that “assuming that both Hecht and decedent desired to conceive a child using decedent’s sperm, real parties fail to establish a state interest sufficient to justify interference with that decision” [18]. This case effectively makes posthumous reproduction with written consent legal in California. This need for written consent was later upheld in California in *Estate of Kievernagel* (2008) and *Robertson v. Saadat* (2020) [20].

*The Matter of Daniel Thomas Christy* (2007) was a case that originated when Daniel Christy was declared dead by neurological criteria after a motorcycle accident in Iowa. His fiancée, with the consent of his parents, requested posthumous sperm retrieval so she could potentially conceive his child. The hospital refused to conduct this procedure absent of a court order, prompting the family to seek judicial authorization. Daniel was an organ donor, so the Iowa District Court ruled that under the Uniform Anatomical Gift Act (UAGA), since sperm can be defined as “tissue”, the sperm retrieval can be considered an anatomical gift, stating that “under the act, an anatomical gift, including the gift of semen, can be made by the donor, or, if the donor did not refuse to make the gift, by the donor’s parents following the donor’s death” [20,21]. This is similar to Australian law [22] and arguments made by legal scholars in the United States [23]. If gametes are able to be considered anatomical gifts, then retrieval should be permissible as long as the deceased person is an organ donor.

*In re Matter of Zhu* (2019) was a case in New York where Peter Zhu, a 21-year-old cadet, faced severe spinal injuries in a skiing accident. Since he was an organ donor, when he was declared dead by neurological criteria, he was kept on somatic support until the scheduled date for organ removal. His parents filed a motion to posthumously retrieve sperm since Peter had periodically expressed his desire to have children. Due to the quick turnaround of this case on the procedure (sperm must be retrieved within 24–48 h after death), the court divided this case into two separate decisions: (i) retrieving the sperm and (ii) approving the use of the retrieved sperm. The court ended up allowing the sperm retrieval to happen and then later granted Peter’s parents full discretion over the utilization of the sperm, including in procreative uses [24]. Despite the court’s argument, this decision faced a lot of backlash for straying from previous precedent. Critics argued that since this request was made by his parents and not a spousal or romantic partner and because there was no written consent from Peter to have his sperm retrieved, the existing statutes are inapplicable to circumstances such as those present in this case; they called for clear legislation on posthumous reproduction to aid courts in making such decisions [25].

It is important to note that this constitutional framework presents significant tensions when applied to posthumous contexts. The cases establishing reproductive rights address living individuals making autonomous choices about their own bodies and reproductive futures. Posthumous reproduction introduces a categorically different scenario: a deceased person who cannot consent, revoke consent, or exercise ongoing decision-making, with living parties making claims about the deceased’s hypothetical wishes. These tensions become even more pronounced in cases involving minors, who cannot have established the kind of adult reproductive intentions that courts in cases like Hecht and Zhu found persuasive. Due to the lack of any clear answer from the Courts, discretion happens more locally and individually by case, e.g., sperm banks, bedside clinicians, and their respective organizations. The next section will explore national healthcare guidelines in the United States with regard to posthumous reproduction.

### 5.4. National Healthcare and Organizational Guidelines

With the lack of legal foundations for posthumous reproduction, local health systems and national professional organizations have stepped in to fill that gap. An often-referenced guideline is the *Weill Cornell Medical Center at NewYork-Presbyterian (NY-P) Hospital* guidelines. NY-P only considers posthumous sperm retrieval requests made from the decedent’s spouse and has no mention of oocyte retrieval. The request must be accompanied by ample evidence that the decedent wanted children and would have wanted to conceive children posthumously; the request also must have unanimous agreement among other members of the immediate family. The spouse has full discretion over the sperm and is also the only person with whom the sperm can be used for procreation. Medical requirements for the retrieval include conducting the procedure no more than 24 h after death, and the decedent must have no communicable disease and no adverse medical circumstances, including HIV, hepatitis, and genetic conditions. They recommend a one-year quarantine with psychological counseling before IVF (for bereavement reasons) and do not indicate that such procedures will be provided at their facilities [26].

On the West Coast, *Stanford Healthcare* created guidelines for its practitioners and institutions after an empirical analysis of existing posthumous reproduction procedures around the United States. They established two separate protocols. The first protocol dictates that the request for sperm retrieval not be granted unless those who request the procedure can prove that the deceased consented to it before his death. This can be through a notarized written directive that indicates (a) authorization of posthumous sperm retrieval and (b) who is permitted to receive the sperm. There is also a requirement that the person receiving the sperm locate a storage facility and indicate who is to provide financial compensation for cryopreservation. The second protocol does not require written consent and instead allows the decedent’s partner to make the sperm retrieval request. It mandates a one-year bereavement period and psychological evaluation and counseling prior to using the retrieved sperm [6]. This policy, again, does not mention posthumous oocyte retrieval.

Finally, the American Society for Reproductive Medicine (ASRM) has a position statement that suggests that decisions and policies regarding posthumous reproduction should be left at the discretion of individual institutions and says that institutions are not at all ethically required to consider posthumous reproduction requests. They further state that institutions should only consider requests made from spouses or other romantic partners who shared a reproductive relationship—they specifically prohibit requests made by parents of the decedent [27,28].

Taken together, the legal and institutional landscape reviewed above tells a consistent story: every existing framework—whether national legislation, court precedent, or hospital policy—was designed with adults in mind, and almost all of it concerns sperm, not eggs. International approaches range from complete prohibition to regulated consent requirements, but none address posthumous oocyte retrieval in minors. U.S. constitutional jurisprudence establishes reproductive rights for living individuals, and even the most permissive case law requires evidence of the deceased’s expressed intent—a standard that is structurally impossible to meet for a minor who died unexpectedly. Hospital guidelines from NY-P and Stanford and the ASRM position statement reinforce this pattern: they set floors around spousal requests, written consent, and bereavement periods, and they explicitly exclude parental requests. None of them contemplate a scenario like Maya Rodriguez’s. The absence of guidance here is not an oversight that can be filled by analogy. It reflects the fact that posthumous oocyte retrieval in a minor presents a categorically different set of ethical problems—around medical burden, consent, relational authority, and child welfare—that require their own analysis. This gap has important public health implications, leaving clinicians without clear guidance, increasing the likelihood of inconsistent decision-making across institutions, and placing undue burden on families and care teams during moments of crisis. The following discussion undertakes that analysis.

## 6. Discussion

Posthumous oocyte retrieval procedures and their ethicality remain undefined and ambiguous in both legal precedent and hospital guidance; further, the dilemma around allowing this procedure in minors is not explicitly addressed. However, posthumous oocyte retrieval in minor girls is disanalogous from both legal and hospital criteria for three main reasons: medical complexity and bodily burden, consent and legal capacity, and spousal requirements.

First, the complexity and time involved in oocyte retrieval procedures is profound. A procedure spanning many weeks of maintaining a minor on somatic supporting measures and tracking ovulatory processes is invasive, expensive, and may pose an undue burden on a minor declared dead by neurological criteria. Following the procedure, the minor will have to be taken off somatic support, raising the concern of limited benefit for the minor from the invasiveness of the oocyte retrieval itself. Most posthumous reproduction law does not even address minors, likely because such cases are exceedingly rare and typically arise under grief-driven or ethically complex parental requests. Moreover, the established precedent is only for posthumous sperm retrieval cases, which are much less invasive and medically less complex as compared with oocyte retrievals. Through a population-level perspective, normalizing such resource-intensive interventions without clear benefit raises concerns about distributive justice, particularly in healthcare systems already marked by disparities in access to care.

Second, due to age restrictions, minors are not allowed to dictate estates or write wills without parental consent. They also cannot provide meaningful advance consent, especially because these cases are likely a result of an unforeseen accident, but also because minors with terminal illness specifying reproductive intentions require parental consent and could be influenced by parental coercion. Assessing assent posthumously is impossible without the minor being able to communicate with healthcare provider teams. Additionally, even if a minor once expressed a desire to have children, their parents may not be a neutral interpreter of that desire. The possibility that a grieving parent projects their own wishes onto a deceased child’s “expressed desires” is ethically serious, and it is arguably more possible for a minor—as opposed to any adult case—because minors have fewer mechanisms to have articulated or documented autonomous reproductive intent. This concern is especially salient in public health research and ethics, which prioritize the protection of vulnerable populations whose ability to exercise autonomy is structurally limited.

Third, all the existing hospital frameworks assume that posthumous reproduction requests are made by adult spouses. This assumption is both too narrow and poorly justified—it excludes other committed relationships that may be equally or more stable than marital ones [29]. Posthumous reproduction standards should instead follow similar vetting standards to those of couples wanting to adopt. Domestic adoption in the United States does not require a marital status. Additionally, although age standards in the United States do vary by state, the youngest an individual is allowed to adopt in any state is at age 18 [30]. Therefore, even if the spousal requirement is broadened to mirror adoptive standards and include those in other committed relationships, minors remain categorically excluded.

Aside from legal and administrative precedent, posthumous oocyte retrieval procedures, especially in minors, introduce a host of new ethical, legal, and practical challenges. First, post-oocyte retrieval surrogacy can be fraught and is currently undefined in United States law and not allowed under peer international law. Next, inheritance law becomes complicated with posthumous reproduction. Current law balances two main dilemmas: the orderly administration of estates given that reproductive material can be stored for years and the decedent’s consent to use of reproductive material [31]. Such issues have already been brought to court; for instance, in *Astrue v. Capato* (2012), the Supreme Court had to determine if twins conceived by in vitro fertilization after their biological father’s death should be considered his “children” under Title II of the Social Security Act (i.e., get benefits). The Supreme Court decided that under the Social Security Act, the twins should not be considered their biological father’s children and denied benefits [32]. Allowing parents to request gamete retrieval for their child will only complicate these processes, introducing an extra generation into the equation where it did not necessarily previously exist.

Legal scholars explicate that posthumous reproduction is legally complicated because it disrupts three core assumptions that undergird inheritance and family law. First, inheritance rules assume that heirs exist or are conceived at the time of death, but posthumously conceived children may not exist or be conceived until months or years later, making it uncertain whether and when they should qualify as heirs. Second, family descent becomes difficult to define because the law must decide whether legal parenthood is based on genetics, formal consent, or reproductive intent—a concept that becomes more complicated when the deceased cannot clarify their wishes. Third, even when states attempt to regulate these situations, the legal framework is fragmented, with inconsistent requirements for consent, timing, and documentation that can lead to different outcomes for similarly situated families across the country. These issues create ambiguity in both who is recognized as a parent and whether posthumously reproduced children are treated as part of the deceased’s family [33].

Lastly, hospital policies, like the one in New York-P, which prohibit posthumous gamete retrieval for individuals who are HIV-positive or carry certain genetic conditions, introduce significant ethical dilemmas. These restrictions not only raise questions about who has the authority to make reproductive decisions for individuals, but they also challenge the principles of autonomy and equality. In the context of posthumous oocyte retrieval, such policies could disproportionately affect marginalized populations, particularly Black and Hispanic communities in the United States, who are more likely to be impacted by HIV. By limiting access to reproductive technologies for these individuals, we risk deepening stigma and reinforcing systemic inequities. These discriminatory guidelines, which imply that certain individuals are ‘unworthy’ of procreation, further complicate the already complex legal and ethical landscape surrounding posthumous reproduction [34]. These patterns highlight how, without clear safeguards and guidelines, emerging reproductive technologies risk reinforcing existing structural disparities—an issue that is central to public health’s commitment to health equity and justice.

An ambiguous political landscape allows violating, unethical posthumous reproduction processes to happen. When the federal law does not draw clear, firm boundaries, cases involving minors can become pivotal and incredibly harmful proving grounds: this may normalize practices that blur individual consent, stretch medical purpose and technology, and allow increasingly coercive interpretations of reproductive intent, both during life and posthumously. Allowing posthumous oocyte retrieval in minors, even in rare cases, risks setting a precedent that erodes established boundaries: medical complexity that can ignore bodily autonomy, age and relationship requirements that disallow confirming consent, and possibilities of coercion, stigma, and legal dilemmas that have the potential to overlook dignity. Ultimately, allowing posthumous oocyte retrieval in minors risks undermining these ethical standards—of bodily autonomy, consent, and dignity—that are central to both reproductive medicine and bioethics.

### Recommendations

The absence of clear guidance governing posthumous oocyte retrieval in minors places an unreasonable burden on bedside clinicians who are asked to make consequential decisions under time pressure, in the presence of grieving families, and without institutional support. Addressing this gap requires action at multiple levels: from individual healthcare institutions, from national professional organizations, and ultimately from legislatures.

At the institutional level, hospitals should adopt explicit policies that establish posthumous oocyte retrieval in minors as a non-permissible procedure by default. These policies should not simply be silent on the question. Silence, as the case of Maya Rodriguez illustrates, functions as ambiguity—and ambiguity in a crisis becomes a bedside decision made under grief. Institutional policy should clearly identify who is authorized to receive posthumous gamete retrieval requests: our recommendation is that the bedside clinician in conjunction with the ethics consultation service establish that requests made by parents or guardians of a deceased minor for oocyte retrieval will be declined and outline a standardized process for communicating this decision compassionately to families. Policies should also provide guidance on documentation and pastoral or psychological support for families whose requests are declined. Institutions with existing posthumous sperm retrieval protocols, such as NewYork-Presbyterian and Stanford, should extend those frameworks to explicitly address oocyte retrieval and to distinguish minor cases from adult cases on procedural grounds.

At the professional society level, the American College of Obstetricians and Gynecologists (ACOG), the American Academy of Pediatrics (AAP), the American Medical Association (AMA), and the American Society for Reproductive Medicine (ASRM) should each publish position statements that address posthumous oocyte retrieval in minors as a distinct and categorically different issue from posthumous sperm retrieval or posthumous reproduction in adults. These statements should, at minimum: (1) affirm that existing adult-centered frameworks for posthumous reproduction cannot be applied to minor decedents without significant modification; (2) articulate the consent, medical burden, and relational authority concerns that distinguish minor cases; and (3) recommend a prohibition on posthumous oocyte retrieval in minors, with any rare exceptions to be evaluated through a formal institutional ethics process—not simply by individual clinicians at the bedside. ASRM in particular should revise its existing position statement on posthumous reproduction to explicitly address parental requests involving minor decedents, closing the gap that its current guidance leaves open. The AAP, given its specific mandate to protect the welfare of minors, is well-positioned to take a leadership role in articulating why minor cases require their own framework rather than an extension of existing adult guidance.

At the legislative level, state legislatures—which have historically been the primary site of regulation for reproductive technology in the absence of federal action—should enact statutes that explicitly address posthumous gamete retrieval in minors. Such statutes should establish age as a threshold criterion, prohibit posthumous oocyte retrieval in individuals under eighteen absent prior written directives executed with parental consent, and define the procedural and relational requirements for any posthumous gamete retrieval request. Federal guidance—whether through legislation or through regulatory action by agencies, such as the Department of Health and Human Services—would provide a more uniform floor than the current patchwork of state law and institutional policy. Until such guidance exists, the default at every level should be restriction, not permission.

Finally, professional ethics bodies and academic medical centers should invest in developing and disseminating educational resources for clinicians who encounter these requests. Ethics consultation teams, reproductive endocrinologists, and palliative care providers are the clinicians most likely to be involved when a request arrives, often within hours of a family’s crisis. They need frameworks for responding that are clear, evidence-based, and ethically grounded—and they need those frameworks before the request arrives, not after.

## 7. Conclusions

Let us return to Maya Rodriguez. Her parents are sitting in a hospital waiting room, freshly devastated, asking a clinical ethics team to keep their daughter on body support for weeks, administer hormonal injections to their brain-dead 17-year-old daughter, retrieve her oocytes through a transvaginal procedure, fertilize them with the sperm of her 18-year-old boyfriend, and arrange a gestational surrogate—all on the basis of a conversation about baby names. The ethics team has no policy. No court has addressed this scenario. No professional organization has published guidance. They are being asked to make this decision at the bedside, under grief, in real time. That is exactly the problem this paper addresses, and why the answer has to be clear: posthumous oocyte retrieval in minor girls should not be permitted. The argument is not that Maya’s parents are acting in bad faith, or that grief makes their request unreasonable. It is that no framework exists capable of honoring this request ethically. Maya could not have provided legally valid consent to posthumous reproductive use of her body. The procedure she would undergo is not brief or minimally invasive: it is weeks of medical intervention on a deceased minor’s body with no direct benefit to her. Additionally, banning this procedure is not about dismissing reproductive rights and autonomy. Rather, it is about recognizing that the ethical preconditions for respecting those rights, capacity, consent, expressed intent, and a framework that accounts for all affected parties cannot be met in this context. Until law and medicine develop the tools to address these cases with the care they require, the appropriate institutional response is to decline requests. Professional medical societies should say so explicitly, hospitals need policies that protect both families and clinicians, and the legislature should not leave these decisions to be made, alone, at the bedside.

## Data Availability

All data used in this review paper is publicly available.
